# Formaldehyde quantification using ampicillin is not selective

**DOI:** 10.1038/s41598-019-54610-3

**Published:** 2019-12-04

**Authors:** Raphael Reinbold, Tobias John, Paolo Spingardi, Akane Kawamura, Amber L. Thompson, Christopher J. Schofield, Richard J. Hopkinson

**Affiliations:** 1 Chemistry Research Laboratory, 12 Mansfield Road, Oxford, OX1 3TA United Kingdom; 2 Ludwig Institute for Cancer Research, Nuffield Department of Medicine, University of Oxford, Oxford, OX3 7DQ United Kingdom; 3 Division of Cardiovascular Medicine, Radcliffe Department of Medicine, Wellcome Trust Centre for Human Genetics, Roosevelt Drive, Oxford, OX3 7BN United Kingdom; 4 Chemical Crystallography, Chemistry Research Laboratory, 12 Mansfield Road, Oxford, OX1 3TA United Kingdom; 5 Leicester Institute of Structural and Chemical Biology and School of Chemistry, University of Leicester, Henry Wellcome Building, Lancaster Road, Leicester, LE1 7RH United Kingdom

**Keywords:** Biochemistry, Cell biology

## Abstract

Formaldehyde (HCHO) is a simple and highly reactive human metabolite but its biochemistry is poorly defined. A limiting factor in HCHO research is lack of validated quantification methods for HCHO relevant to biological samples. We describe spectroscopic studies on a reported fluorescence-based HCHO detection method involving its reaction with ampicillin. The results validate the structure and fluorescence properties of the HCHO-ampicillin reaction product. However, the same adduct is observed after reaction of ampicillin with glyoxylate. Related fluorophores were formed with other biologically relevant carbonyl compounds. Overall, our studies suggest the ampicillin method is not reliable for selective detection and quantification of HCHO in biological samples.

## Introduction

Formaldehyde (HCHO) is an environmental pollutant and an endogenous human metabolite^1^. Exposure to HCHO at elevated concentrations, e.g. from air pollution, food, or cosmetics, is carcinogenic^1^. However, the precise molecular mechanisms underpinning its toxicity are unclear. HCHO is an electrophilic carbonyl compound that undergoes reactions with nucleophilic biomolecules, forming covalent adducts of varying stabilities^1–5^. It is possible such adducts are responsible for HCHO’s toxic/carcinogenic effects, although physiologically relevant studies are lacking.

HCHO is produced in human cells during oxidative demethylation reactions, as catalysed by small molecule amine oxidases and histone and nucleic acid demethylases^6^; other HCHO sources include folate oxidation and serine metabolism^7^. The routine production of HCHO in human cells suggests it has ‘healthy’ functional roles, at least when HCHO concentrations are below toxic levels. Recent pioneering work has identified HCHO as a carbon source in human C1 metabolism and other biological roles are likely^8^.

A limitation in research on HCHO is lack of quantification methods suitable for its analysis in biological samples. Detecting and quantifying HCHO in biological samples is challenging due to its low molecular weight, volatility, and promiscuous, often reversible reactions. Most reported HCHO detection/quantification methods thus employ reagents that form relatively stable adducts with HCHO; detection of the adducts is then achieved by chromatography coupled with fluorescence spectroscopy and/or mass spectrometry^9,10^. Various HCHO scavengers are reported; however, their reactions with HCHO are often incompletely characterised and reversible, implying an inability to fully sequester HCHO.

We are interested in developing methods suitable for HCHO detection in biological research. To this end, we have carried out studies on a reported fluorescence-based HCHO detection method that uses ampicillin, a β-lactam antibiotic, as an HCHO scavenger^11–14^. While this method has been reported to quantify HCHO in human cells^12^, blood^13^ and urine^14^, the structure of the fluorescent adduct has not been fully characterised. It has also been unclear whether the same or similar adducts are formed with other biologically relevant carbonyl compounds.

Our studies on the reaction of ampicillin with carbonyl compounds reveal formation of pyrazinones, differing in their C-6 substituents. Fluorescence analyses revealed near-identical absorbance and emission spectra for all studied pyrazinones, implying selective detection of specific carbonyl compounds by this method requires separation. Further, glyoxylate^15^ reacts with ampicillin to form the same pyrazinone as obtained with HCHO, suggesting the ampicillin method is unable to distinguish between these aldehydes.

## Results

**Figure 1:**
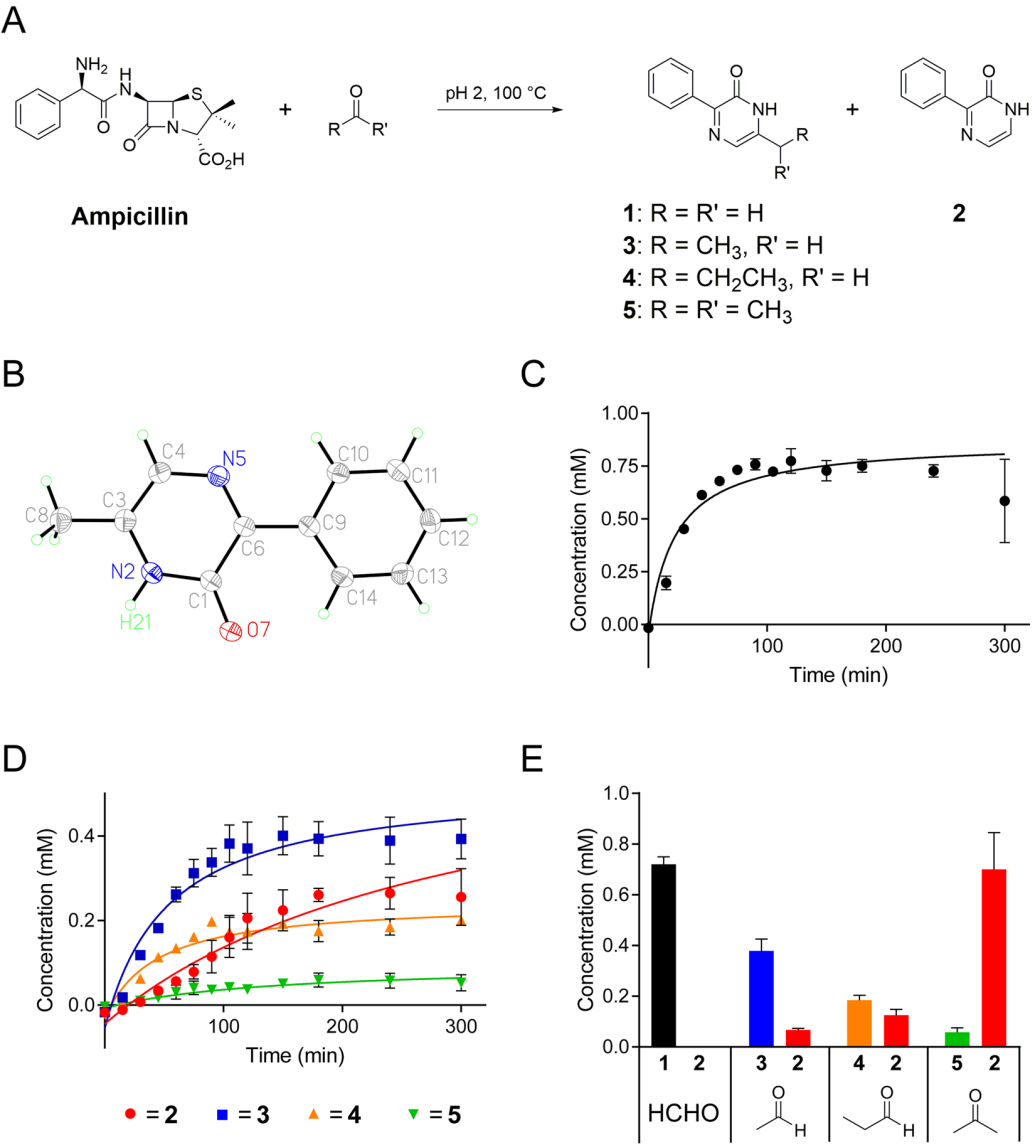
Ampicillin reacts with carbonyl compounds to form 6-substituted 3-phenyl-pyrazin-2-ones. (**A**) Formation of pyrazin-2-ones by reaction of ampicillin with HCHO, acetaldehyde, propionaldehyde, or acetone. The pyrazin-2-one products differ in their C-6 substituents. 2-Phenyl-pyrazin-2-one (**2**) was also observed with acetaldehyde, propionaldehyde, and acetone. Compound **2** had been previously reported as a degradation product of ampicillin^21^. (**B**) Structure of 3-phenyl-6-methyl-pyrazin-2-one (**1**) from reaction of ampicillin and HCHO from single crystal X-ray diffraction studies; displacement ellipsoids drawn at 50% probability. Crystallographic data are deposited in the Cambridge Crystallographic Data Centre (CCDC 1941818). The structure was solved using SuperFlip and refined using CRYSTALS suite (V14.61 Build 7285). Graphics were produced using XP V5.1^16^. (**C**) Time-course of the reaction of ampicillin and HCHO (64 equivalents) (pH 2, 100 °C, 5 hours). Formation of **1** occurs within 2 hours. (**D**) Time-courses showing formation of 6-substituted pyrazin-2-ones in reactions of ampicillin and either acetaldehyde (**3**, blue), propionaldehyde (**4**, orange) or acetone (**5**, green) (pH 2, 100 °C, 5 hours). Note reaction of acetaldehyde, forming **3**, is the most efficient. Formation of **2** from ampicillin (without carbonyl substrate) also occurs (red, note: **2** is also formed in other reactions, see (**E**)). (**E**) Chart showing concentrations of pyrazin-2-ones in reactions of ampicillin and either HCHO, acetaldehyde, propionaldehyde, or acetone (64 equiv.) after 2 hours (pH 2, 100 °C). **2** was not observed with HCHO, but was observed with acetaldehyde, propionaldehyde, and acetone.

Initially, we focused on characterising the structure of the ampicillin- and HCHO-derived fluorophore. As in prior studies^12–14^, ampicillin was added to HCHO (21 equiv.) in H_2_O, then the mixture was acidified (pH 2). After 16 hours incubation at 100 °C, a yellow solution was obtained; extraction into ethyl acetate followed by evaporation gave a yellow solid. Analysis by liquid chromatography–mass spectrometry (LC/MS) indicated the presence of two species, which were separated by flash chromatography. NMR analyses on the isolated compounds revealed them both to be 3-phenyl-pyrazin-2-ones (Fig. 1A). The major isolated species (**1**) contained a methyl substituent, which was assigned to the 6-position of the pyrazinone ring on the basis of 2-dimensional ^1^H-^13^C-heteronuclear multiple bond correlations (HMBC). This assignment (3-phenyl-6-methyl-pyrazin-2-one) was further supported by single crystal X-ray diffraction studies (Fig. 1B)^16^. The minor species, **2**, present at substantially lower levels, lacked the C-6 methyl substituent (3-phenyl-pyrazin-2-one).

We then conducted time-course analyses on the reaction of ampicillin and HCHO. A solution of ampicillin in H_2_O was reacted with HCHO (64 equiv.) at pH 2, then incubated at 100 °C for 5 hours. Aliquots were taken at 15, 30, 45, 60, 75, 90, 105, 120, 150, 180, 240 and 300 minutes and analysed by LC/MS; compound concentrations were determined by comparing the signal intensities in the LC/MS spectra with those of purified controls. The time-course analyses revealed that **1** is slow to form, reaching a maximum concentration of 775 μM after two hours; no evidence for formation of **2** was accrued by LC/MS, suggesting preferential formation of **1** over **2** under these tested conditions (Fig. 1C).

We then monitored the reactions of ampicillin with acetaldehyde, propionaldehyde and acetone. In all cases, two species were observed; one species was present in all samples and was assigned as **2**; this suggests that the formation of **2** is independent of the tested carbonyl reactants. The acetaldehyde, propionaldehyde and acetone product mixtures also contained a 6-substituted 3-phenyl-pyrazin-2-one ring. These were assigned on the basis of NMR and MS analyses as 3-phenyl-6-ethyl-pyrazin-2-one, **3**, 3-phenyl-6-propyl-pyrazin-2-one, **4**, and 3-phenyl-6-isopropyl-pyrazin-2-one, **5**, respectively. Time-course studies, conducted under identical conditions to the studies with HCHO, revealed the 6-substituted pyrazinones to be the major isolated products for the reactions with acetaldehyde and propionaldehyde (Figs. 1D,E and S1). However, the maximum concentrations of both **3** (400 μM) and **4** (200 μM) were lower than observed for **1** with HCHO (775 μM), while the concentrations of **2** in the reactions inversely correlated with those of the 6-substituted pyrazinones (Fig. 1E). With acetone, the 6-substituted pyrazinone **5** only reached a maximum concentration of 60 μM; formation of **2** was higher reaching a maximum of 700 μM after two hours. In the absence of any carbonyl containing substrate, only formation of **2** was observed. In some time-course experiments, including in the time-course with HCHO, a decrease in the pyrazinone concentrations was observed at later time points. This observation may be evidence for further reactions involving the pyrazinones; however, we could not identify products from such reactions by LC/MS analysis.

**Figure 2:**
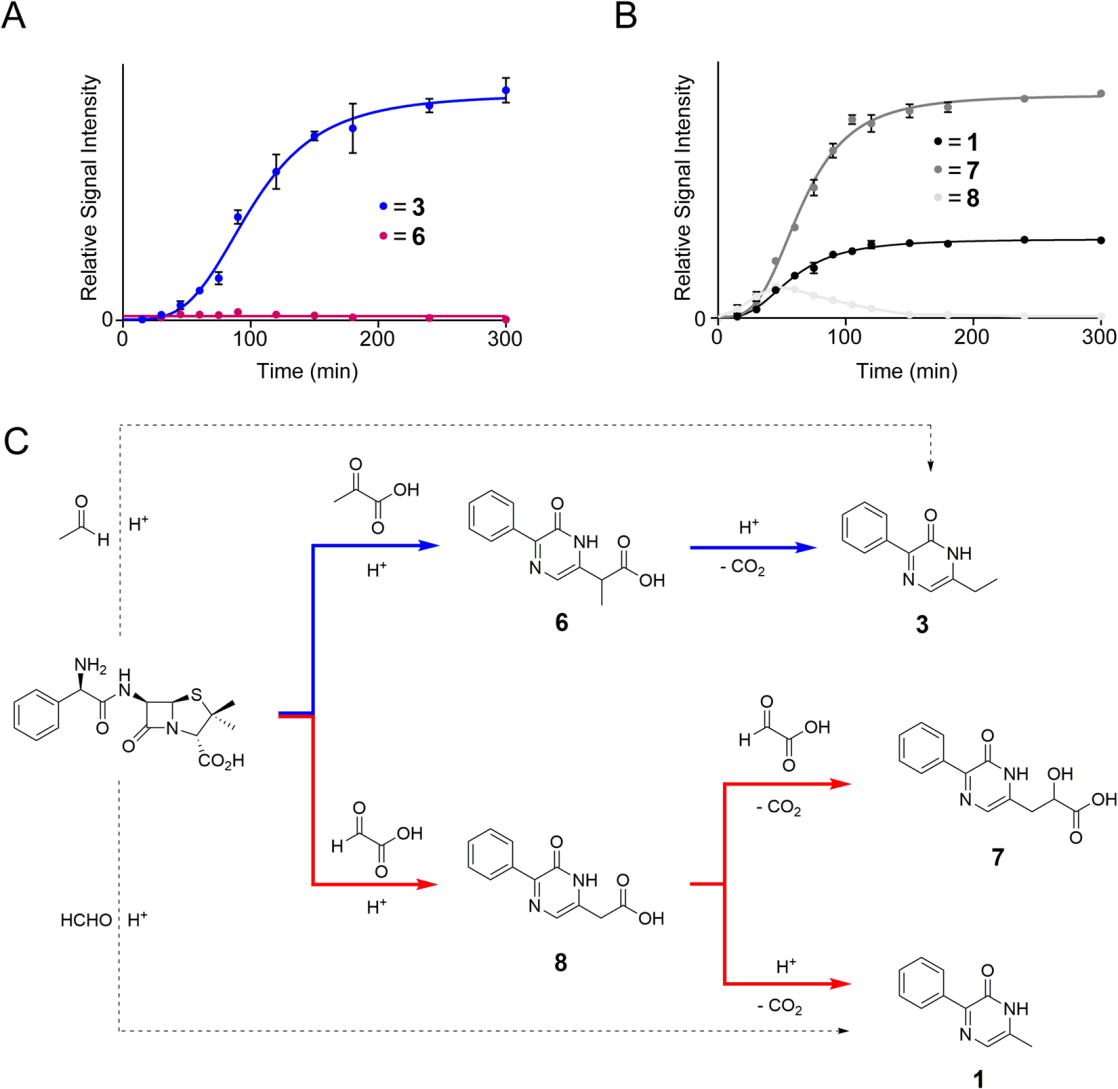
Reactions of ampicillin with pyruvate and glyoxylate lead to multiple 6-substituted 3-phenyl-pyrazin-2-ones. (**A**) Time-course of the reaction of ampicillin and pyruvate (64 equiv.) (pH 2, 100 °C, over 5 hours). The major product is 3-phenyl-6-ethyl-pyrazin-2-one (**3**) which is also formed by reaction of ampicillin with acetaldehyde. Low level production of **6** is also observed. (**B**) Time-course of the reaction of ampicillin and glyoxylate (64 equiv.) (pH 2, 100 °C, over 5 hours). At early time points, **1**, **7** and **8** are observed; after 3 hours, only **1** and **7** are observed. (**C**) Scheme showing proposed formation pathways of pyrazinones **1**, **3**, **6**, **7** and **8** in reactions of ampicillin with pyruvate and glyoxylate.

We then analysed reactions of ampicillin with pyruvate and glyoxylate, which are human metabolites produced during glycolysis and glycine metabolism, respectively^15^. Samples containing ampicillin and either pyruvate or glyoxylate (64 equiv.) were prepared as previously described, then analysed over 5 hours by LC/MS. Interestingly, formation of **2** was not observed in these samples; however, multiple other species were observed. With pyruvate, two compounds were detected by LC/MS, the major being **3**, i.e. the same product observed in the reaction with acetaldehyde (Fig. 2A,C). The other compound, which was only present at trace levels, was assigned as 2-(6-oxo-5-phenyl-1,6-dihydropyrazin-2-yl)propanoic acid, **6**, on the basis of MS and NMR analyses (Fig. 2A,C). The structure of **6** resembles **3** but contains an additional carboxylate, suggesting it may be an intermediate in the formation of **3**.

**Figure 3:**
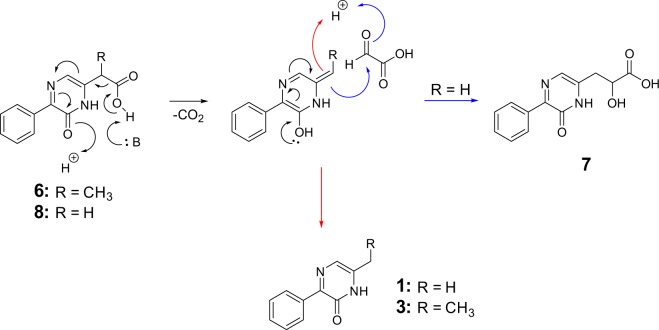
Proposed mechanism for the formation of **1**, **3** and **7** from carboxylated pyrazin-2-ones. Decarboxylation of **6** and **8** gives intermediates that can tautomerise (red) to form **1** and **3**. The intermediate derived from **8** can react with another molecule of glyoxylate to form **7** (blue).

With glyoxylate, three extracted species were observed (Fig. 2B,C). The major product was assigned as 2-hydroxy-3-(6-oxo-5-phenyl-1,6-dihydropyrazin-2-yl)propanoic acid, **7**; the 2-hydroxy-propanoate substituent at C-6 of its pyrazinone ring likely derives from two glyoxylate molecules. The least abundant species was assigned as 2-(6-oxo-5-phenyl-1,6-dihydropyrazin-2-yl)acetic acid, **8**, which has a 2-carboxy-ethyl substituent at C-6. Importantly, the second most abundant pyrazinone observed was **1**, i.e. the same pyrazinone observed after reaction with HCHO. As proposed for the formation of **3** from pyruvate, it is likely **1** is formed by decarboxylation of **8** followed by protonation, while the anionic decarboxylated intermediate could react with another glyoxylate, forming **7** (Fig. 3). This proposal is supported by time-course analyses, which reveal initial formation of **8** before its depletion concomitant with formation of **1** and **7** (Fig. 2B).

Finally, we determined the absorbance spectra of the isolated pyrazinones **1**-**8**. All species exhibited similar absorbance spectra with one major absorbance band; the wavelength of maximum absorbance for each band was within 11 nm (340-351 nm, Figs. S2 and S3). We then collected emission spectra for each compound under excitation at 347 nm. In all cases, the spectra exhibited near-identical line-shapes, with maximal emission wavelengths within 30 nm (Figs. S2 and S3). Overall, these studies reveal the pyrazinones **1**-**8** have similar absorbance/fluorescence properties.

## Discussion

Studies on the reactions of ampicillin and HCHO under acidic conditions reveal formation of a 3-phenyl-pyrazin-2-one adduct (**1**) detectable by MS and/or fluorescence spectroscopy. Similar 3-phenyl-pyrazin-2-ones (**2**-**8**) were observed in reactions of ampicillin with other carbonyl compounds, and in the absence of carbonyl substrate, implying a lack of selectivity of ampicillin for HCHO. Time-course analyses reveal pyrazinone formation is slow under the reported conditions; of the tested carbonyls, HCHO and acetaldehyde reacted most efficiently, forming **1** and **3** respectively, although reaction completion was only observed after 2 hours. Further, formation of **2** was observed in samples with acetaldehyde, propionaldehyde and acetone, while samples with pyruvate or glyoxylate formed multiple pyrazinones, including **3** and **1**, respectively.

**Figure 4:**
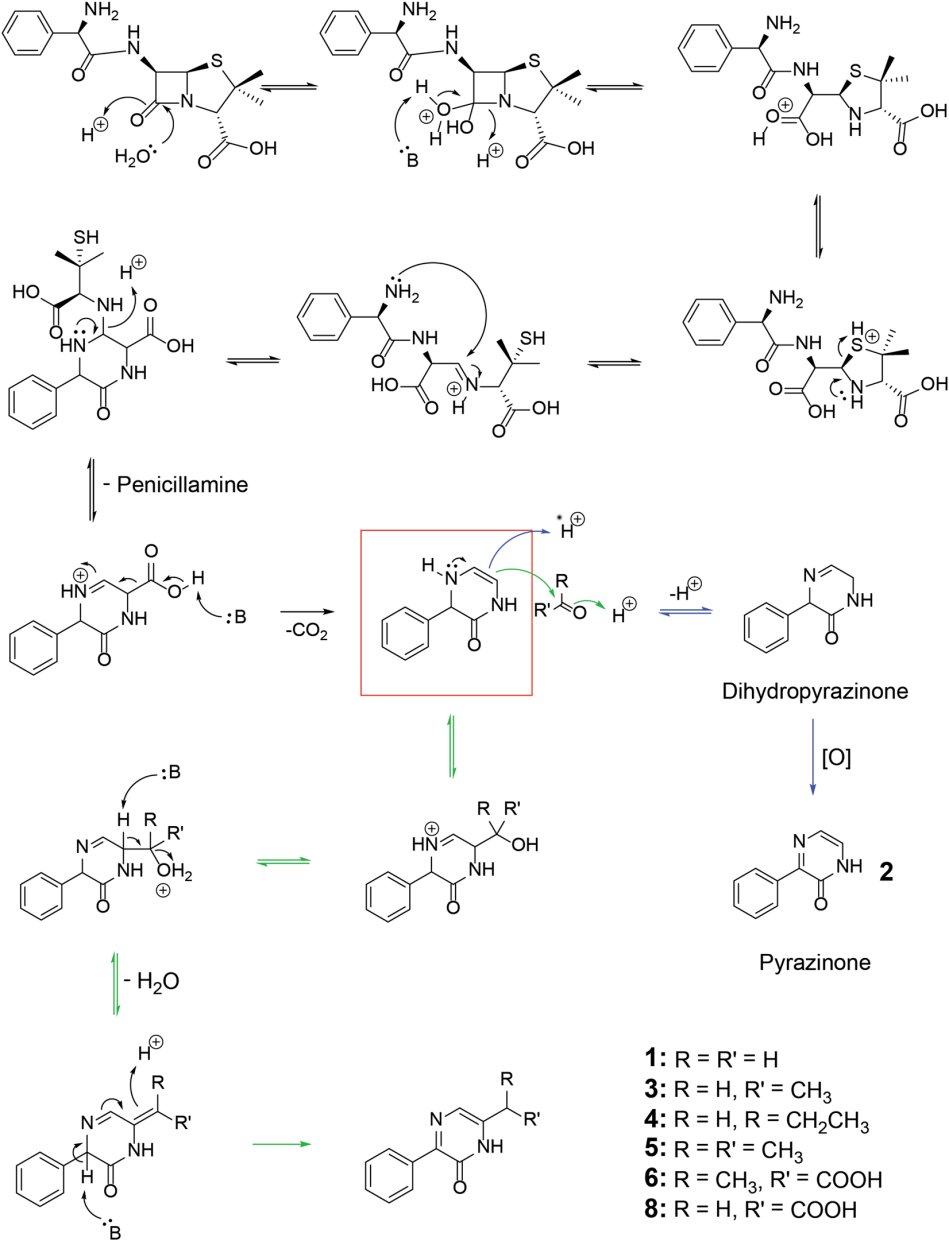
Proposed mechanism for the formation of pyrazin-2-ones **1**, **3**, **4**, **5**, **6** and **8** by reaction of ampicillin with carbonyl compounds. Ampicillin degrades in acidic conditions to form an enamine (red box) that reacts with electrophilic carbonyls (green arrows). Elimination of water then gives the pyrazinone product. Competing protonation, followed by oxidation (blue arrows), gives pyrazinone **2**.

The observed differences in reaction efficiencies, as well as the 6-substituents in the products, suggest a conserved general mechanism for pyrazinone formation (Fig. 4). A key step in this mechanism is the reaction of the electrophilic carbonyl group with a 3,4-dihydro-pyrazin-2-one intermediate, nucleophilic at C-6 (red box, Fig. 4). Condensation of the resultant hydroxylated product gives a pyrazinone ring, with the nature of the C-6 substituent depending on the carbonyl substrate (**1**, **3**-**5**). Competing protonation gives **2** after oxidation of the intermediate dihydropyrazinone; for less electrophilic carbonyl substrates, e.g. propionaldehyde/acetone, protonation to give **2**, a process with proposed precedence in pyrazinone biosynthesis^17^, better competes with aldol type reactions (Fig. 1E). For pyruvate and glyoxylate, reactions with the 3,4-dihydro-pyrazin-2-one intermediate result in pyrazinones susceptible to irreversible decarboxylation (**6** and **8**, Fig. 3); the resultant decarboxylated intermediates are then either protonated (forming **1** or **3**) or, in the case of glyoxylate, susceptible to further reaction with the carbonyl substrate, forming **7** (Fig. 3).

The combined results suggest the ampicillin method can be used to sequester multiple carbonyl compounds from aqueous mixtures, and that the resultant pyrazinones can be analysed using fluorescence spectroscopy. However, because of the similar fluorescence properties of the pyrazinones, selective detection necessitates (chromatographic) separation before analysis. The identification of ‘HCHO-derived’ pyrazinone **1** from reaction with glyoxylate reveals the method is unsuitable for distinguishing between these metabolites, indicating the method is insufficiently selective for quantifying HCHO in complex biological samples. The findings also further illustrate the potential for complexity in the reactions of HCHO and other reactive carbonyl compounds in reactions with biomolecules and drugs^1–5,18^, and will be useful for ongoing method development on methods for analysing HCHO and other endogenous reactive carbonyl compounds. It is also notable that some clinically-relevant β-lactams, e.g. sultamicillin^19^ and metampicillin^20^, are administered as prodrugs that likely fragment to give the active antibiotic and HCHO.

## Methods

### Synthesis of pyrazin-2-ones for characterisation

Ampicillin sodium salt (199 mg, 0.54 mmol) was dissolved in water (40 mL) and the solution was acidified to pH 2. The corresponding carbonyl compound (11.46 mmol) was then added and the mixture was refluxed overnight at 100 °C. After extraction with ethyl acetate, the product was purified by silica flash chromatography. Synthesis of compound **5** from ampicillin and acetone was conducted in a microwave reactor (Biotage) in a sealed reaction vessel (10 mL, 100 °C, 30 minutes) to avoid loss of volatile acetone during reflux. Syntheses of compounds **6** and **8** from ampicillin and pyruvate and glyoxylate respectively were conducted at 60 °C overnight to reduce the extent of decarboxylation.

### Time-course studies

The ampicillin sodium salt (150 mg, 0.4 mmol) and the carbonyl compound (8.6 mmol) were dissolved in H_2_O (30 mL) and the solution was acidified to pH 2. The solution was then aliquoted into three microwave reaction vessels (10 mL per vessel). Each vessel was then heated in heating block at 100 °C with stirring. Samples were taken and immediately frozen on dry ice after 0 min (before heating) and after 15 minutes, 30 minutes, 45 minutes, 60 minutes, 75 minutes, 90 minutes, 105 minutes, 2 hours, 2.5 hours, 3 hours, 4 hours, and 5 hours heating. The samples were then analysed by LC/MS using an Agilent Technologies 1200 series LC/MS system with an H_2_O/MeCN solvent system (if a precipitate formed during the reaction, the samples were diluted 1:1 with acetonitrile (MeCN) to dissolve the precipitate before analysis). MS signal intensities for each product were extracted from the spectra and were calibrated using purified controls (products **1**-**5**). A control time-course without added carbonyl was also conducted, which revealed formation of **2**.

### Absorbance and Fluorescence Studies

Samples were dissolved (30 mM) in a 1:1 mixture of H_2_O (pH 2) and MeCN and then diluted in to a final volume of 50 µL (final concentration = 2 µM). Absorbance spectra were measured on the diluted samples between 280 and 450 nm using a Clariostar plate reader (BMG Labtech). For fluorescence measurements, an excitation wavelength of 347 nm (range: 10 nm) was selected to ensure strong excitation. The fluorescence emission spectra were measured between 365 and 550 nm.

## Supplementary information

Supplementary Information
