# Supplementary material for: Formaldehyde quantification using ampicillin is not selective
Source: Sci Rep. 2019 Dec 4;9:18289. doi: 10.1038/s41598-019-54610-3 (PMC6892939; doi:10.1038/s41598-019-54610-3)
Supplement: Supplementary file 1 — Supplementary Information [file 41598_2019_54610_MOESM1_ESM.pdf]

Supplementary Information

Formaldehyde quantification using ampicillin is not selective

Raphael Reinbold,[a] Tobias John,[a] Paolo Spingardi,[a,b] Akane Kawamura,[a,c] Amber L. Thompson,[d] Christopher J. Schofield,*[a] and Richard J. Hopkinson*[a,e]

[a]Chemistry Research Laboratory, 12 Mansfield Road, Oxford, OX1 3TA, United Kingdom. [b]Ludwig Institute for Cancer Research, Nuffield Department of Medicine, University of Oxford, Oxford, OX3 7DQ, United Kingdom. [c]Division of Cardiovascular Medicine, Radcliffe Department of Medicine, Wellcome Trust Centre for Human Genetics, Roosevelt Drive, Oxford OX3 7BN, United Kingdom. [d]Chemical Crystallography, Chemistry Research Laboratory, 12 Mansfield Road, Oxford, OX1 3TA, United Kingdom. [e]Leicester Institute of Structural and Chemical Biology and Department of Chemistry, University of Leicester, Henry Wellcome Building, Lancaster Road, Leicester, LE1 7RH, United Kingdom. E-mail: richard.hopkinson@leicester.ac.uk

Reagents

Starting materials were from Sigma Aldrich and Alfar Aesar.

Characterisation Methods

NMR spectra were recorded using Bruker Avance AV400 or AV500 machines. The Bruker internal referencing procedure (edlock) was used to reference the spectra to the solvent peak. Coupling constants (J) are reported to the nearest 0.5 Hz. Some ^{13}C resonances were not observed in ^{13}C NMR spectra but were observed in 2-dimensional ^{1}H-^{13}C heteronuclear single quantum coherence (HSQC) spectra. Mass spectra were collected using an Agilent single quadrupole machine. High-resolution mass spectra measurements were conducted on a Waters LCT Premier ESI mass spectrometer. Melting points were measured using a Stuart automatic melting point SMP40 with a heating rate of 20 °C per minute. Infrared (IR) spectra were recorded on a Bruker Tensor 27 FT-IR spectrometer using ATR technology at room temperature.

Compound Characterisation

Compounds were synthesised according to *Synthesis of pyrazin-2-ones for characterisation* (Main text, Method section).

3-Phenyl-6-methyl-pyrazin-2-one, 1

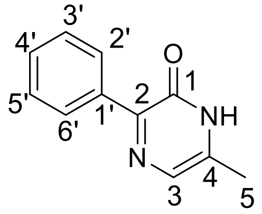

A yellow solution was obtained. Purification by silica flash chromatography yielded a yellow solid (37.5 mg, 0.2003 mmol, 37 %).

IR ṽ 1647.3 cm^{-1}, 1614.7 cm^{-1} (CONH); ^{1}H-NMR (500 MHz, d_6-DMSO): δ (ppm) 12.52 (s, 1H, C1-N*H*), 8.26-8.28 (m, 2H, C2'-*H*/C6'-*H*), 7.39-7.43 (m, 3H, C3'-*H*/C4'-*H*/C5'-*H*), 7.33 (s, 1H, C3-*H*), 2.21 (s, 3H, C5-*H*); ^{13}C-NMR (125 MHz, d_6-DMSO): δ (ppm) 155.72 (C1), 147.93 (C2), 137.53 (C4), 136.21 (C1'), 128.95 (C4'), 127.98 (C3', C5'), 127.78 (C2', C6'), 121.94 (C3), 15.33 (C5), HRMS (FI+) m/z: calculated for $C_{11}H_{11}N_2O$ $(M+H)^+$: 187.08659, found 187.08662; Melting point: 212 °C**.**

3-Phenyl-pyrazin-2-one, 2

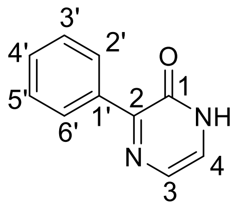

A yellow solution was obtained. Purification by silica flash chromatography yielded a yellow solid (26.6 mg; 0.1245 mmol, 29 %).

IR ṽ 1639.6 cm^{-1}, 1594.4 cm^{-1} (CONH); ^{1}H-NMR (500 MHz, d_6-DMSO): δ (ppm) 12.48 (s, 1H, C1-N*H*), 8.27-8.29 (m, 2 H, C2'-*H*/C6'-*H*), 7.47 (d, 1H, C4-*H*, J= 4.0 Hz), 7.42-7.44 (m, 4 H, C3-*H*/C3'-*H*/C4'-*H*/C5'-*H*); ^{13}C-NMR (125 MHz, d_6-DMSO): δ (ppm) 155.38 (C1), 151.57 (C2), 136.08 (C1'), 129.43 (C4'), 128.27 (C3', C5'), 127.83 (C2' ,C6'), 126.94 (C3), 123.13 (C4); HRMS (FI+) m/z: calculated for $C_{10}H_9N_2O$ $(M+H)^+$: 173.07094, found 173.07108; Melting point: 171 °C.

3-Phenyl-6-ethyl-pyrazin-2-one, 3

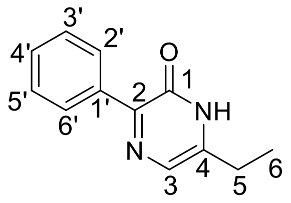

A yellow solution was obtained. Purification by silica flash chromatography yielded a yellow solid (28.9 mg, 0.1443 mmol, 26 %).

IR ṽ1630.7 cm^{-1}, 1600.7 cm^{-1} (CONH); ^{1}H-NMR (500 MHz, d_6-DMSO): δ (ppm) 12.51 (s, 1H, NH), 8.26-8.28 (m, 2H, C2'-*H*/C6'-*H*), 7.36-7.44 (m, 4H, C3-*H*, C3'-*H*/C4'-*H*/C5'-*H*), 2.49-2.54 (q, 2H, C5-*H*, J=7.5 Hz; signal slightly overlapped by solvent peak), 1.21 (t, 3H, C6-*H*, J= 7.5 Hz); ^{13}C-NMR (125 MHz, d_6-DMSO): δ (ppm) 155.84 (C1), 148.32 (C2), 142.52 (C4), 136.21 (C1'), 129.00 (C4'), 128.04 (C3', C5'), 127.80 (C2', C6'), 120.81 (C3), 23.02 (C5), 12.85 (C6); HRMS (FI+) m/z: calculated for $C_{12}H_{13}N_2O$ $(M+H)^+$: 201.10224, found 201.10231; Melting point: 168 °C.

3-Phenyl-6-propyl-pyrazin-2-one, 4

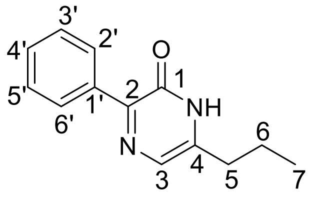

A yellow solution was obtained. Purification by silica flash chromatography yielded a yellow solid (36.2 mg, 0.1689 mmol, 31 %).

IR ṽ 1632.7 cm^{-1}, 1598.4 cm^{-1} (CONH); ^{1}H-NMR (500 MHz, d_6-DMSO): δ (ppm) 12.49 (s, 1H, NH), 8.27-8.29 (m, 2H, C2'-*H*/C6'-*H*), 7.39-7.43 (m, 4H, C3-*H*, C3'-*H*/C4'-*H*/C5'-*H*), 2.47 (t, 2H, C5-*H*, J= 7.5Hz), 1.64 (sext., 2H, C6-*H*, J= 7.5Hz), 0.91 (t, 3H, C7-*H*, J= 7.5Hz); ^{13}C-NMR (125 MHz, d_6-DMSO): δ (ppm) 155.90 (C1), 148.72 (C2), 141.08 (C4), 136.20 (C1'), 128.99 (C4'), 128.02 (C3', C5'), 127.78 (C2', C6'), 121.60 (C3), 31.40 (C5), 21.45 (C6), 13.36 (C7); HRMS (FI+) m/z: calculated for $C_{13}H_{15}N_2O$ $(M+H)^+$: 215.11789, found: 215.11802; Melting point: 185 °C.

3-Phenyl-6-isopropyl-pyrazin-2-one, 5

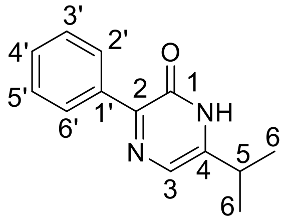

A yellow solution was obtained. Purification by silica flash chromatography yielded a yellow solid (12.5 mg, 0.05831 mmol, 11 %).

IR ṽ 1637.2 cm^{-1}, 1598.7 cm^{-1} (CONH); ^{1}H-NMR (500 MHz, d_6-DMSO): δ (ppm) 12.47 (s, 1H, NH), 8.25-8.27 (m, 2H, C2'-*H*/C6'-*H*), 7.38-7.44 (m, 4H, C3-*H*, C3'-*H*/C4'-*H*/C5'-*H*), 2.82 (sept., 1H, J= 7 Hz, C5-*H*) ,1.25 (d, 6H, J= 7Hz, C6-*H);* ^{13}C-NMR (125 MHz, d_6-DMSO): δ (ppm) 156.37 (C1), 149.23 (C2), 146.52 (C4), 136.65 (C1'), 129.49 (C4'), 128.54 (C3', C5'), 128.28 (C2', C6'), 119.69 (C3), 29.81 (C5), 21.46 (2 x C6); HRMS (FI+): m/z: calculated for $C_{13}H_{15}N_2O$ $(M+H)^+$: 215.11789, found: 215.11791; Melting point: 156 °C.

2-(6-Oxo-5-phenyl-1,6-dihydropyrazin-2-yl)propanoic acid, 6

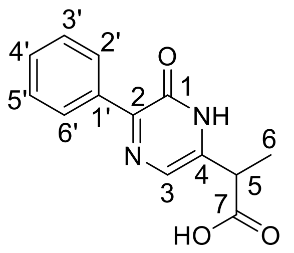

A yellow solution was obtained. Purification by silica flash chromatography yielded a yellow solid (13.3 mg, 0.0545 mmol, yield: 10 %).

IR ṽ 1721.1 cm^{-1}, 1640.4 cm^{-1} (CONH); ^{1}H-NMR (500 MHz, d_6-DMSO): δ (ppm) 12.61 (s, 2H, CONH and COOH), 8.20-8.28 (m, 2H, C2'-*H*/C6'-*H*), 7.39-7.46 (m, 4H, C3-*H*, C3'-*H*/C4'-*H*/C5'-*H*), 3.74 (q, 1H, J= 7.5 Hz, C5-*H*), 1.47 (d, 3H, J= 7.5 Hz, C6-*H);* ^{13}C-NMR (125 MHz, d_6-DMSO): δ (ppm) 173.45 (C7) 156.41

(C1), 147.87 (C2), 141.13(C4), 136.44 (C1’), 129.67 (C4’), 128.72 (C3’, C5’), 128.38 (C2’, C6’), 120.89 (C3), 40.78 (C5), 15.79 (C6); HRMS (FI+): m/z: calculated for $C_{13}H_{13}N_2O_3$ $(M+H)^+$: 245.09207, found: 245.09174; Melting point: 140 °C.

2-Hydroxy-3-(6-oxo-5-phenyl-1,6-dihydropyrazin-2-yl)propanoic acid, 7

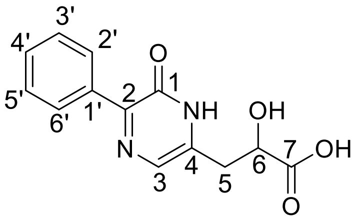

An orange solution was obtained. Purification by silica flash chromatography yielded an orange solid. (46.1 mg, 0.177 mmol, 33 %).

IR ṽ 1729.3 cm^{-1}, 1638.6 cm^{-1} (CONH); ^{1}H-NMR (500 MHz, d_4-Methanol): δ (ppm) 8.14-8.19 (m, 2H, C2’-*H*/C6’-*H*), 7.47 (s, 1H, C3-*H*), 7.39-7.46 (m, 3H, C3’-*H*/C4’-*H*/C5’-*H*), 4.49 (dd, 1H, C6-*H*, J=4.5 Hz; 9 Hz), 3.10 (dd, 1H, C5-*H*, J=4.5 Hz, 14.5 Hz), 2.90 (dd, 1H, C5-*H*, J= 9 Hz, 14.5 Hz); ^{13}C-NMR (125 MHz, d_6-DMSO): δ (ppm) 173.06 (C7), 154.93 (C1), 149.01 (C2), 135.93 (C4), 134.29 (C1’), 127.7 (C4’), 126.73 (C3’, C5’), 126.05 (C2’, C6’), 122.55 (C3), 67.43 (C10), 32.98 (C9); HRMS (FI+) m/z: calculated for $C_{13}H_{13}O_4N_2$ $(M+H)^+$: 261.08698, found: 261.08714; Melting point: 178 °C.

2-(6-Oxo-5-phenyl-1,6-dihydropyrazin-2-yl)acetic acid, 8

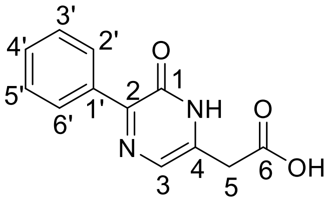

Due to side reactions a reaction to obtain significant amounts of compound 8 was carried out at 60 °C. A yellow solution was obtained. Purification by silica flash chromatography yielded an orange solid. (30.65 mg, 0.133 mmol, 25%)

IR ṽ 1653.9 cm^{-1}, 1525.6 cm^{-1} (CONH); ^{1}H-NMR (500 MHz, d_6-DMSO): δ (ppm) 12.63 (s, 1H, NH), 8.25-8.28 (m, 2H, C2’-*H*/C6’-*H*), 7.42-7.45 (m, 4H, C3-*H*, C3’-*H*/C4’-*H*/C5’-*H*), 3.59 (s, 2H, C5-*H*); ^{13}C-NMR (125 MHz, d_6-DMSO): δ (ppm) 171.04 (C6), 155.71 (C1), 148.07 (C2), 137.38 (C4), 136.20 (C1’), 128.96 (C4’), 127.98 (C3’, C5’), 127.79 (C2’, C6’), 121.78 (C3), 35.33 (C5); HRMS (FI+) m/z: calculated for $C_{13}H_{15}N_2O$ $(M+H)^+$: 231.07642, found: 231.07669; Melting point: degraded (166 °C).

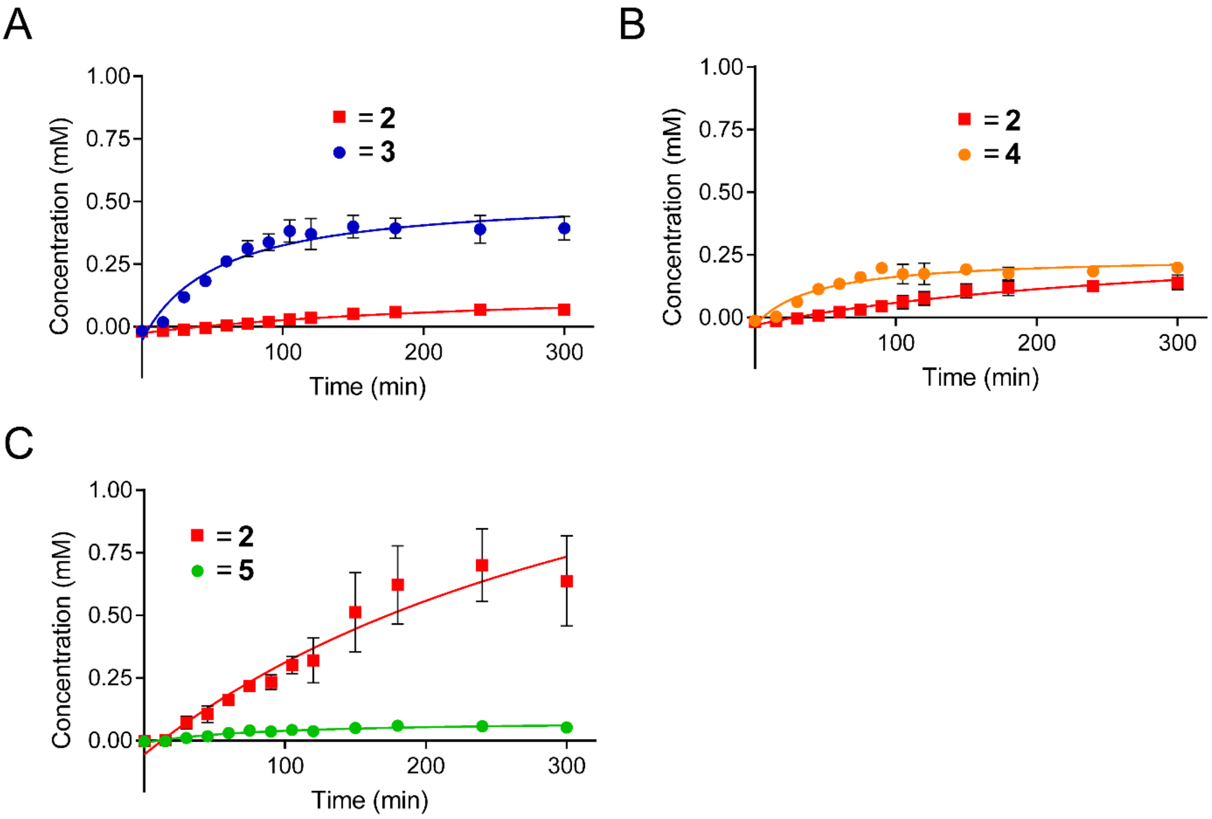

Figure S1. (A) Time-course of the reaction of ampicillin and acetaldehyde (64 equivalents) at pH 2 and 100 °C over 5 hours. Formation of **2** and **3** is observed. (B) Time-course of the reaction of ampicillin and propionaldehyde (64 equivalents) at pH 2 and 100 °C over 5 hours. Formation of **2** and **4** is observed. (C) Time-course of the reaction of ampicillin and acetone (64 equivalents) at pH 2 and 100 °C over 5 hours. Formation of **2** and **5** is observed.

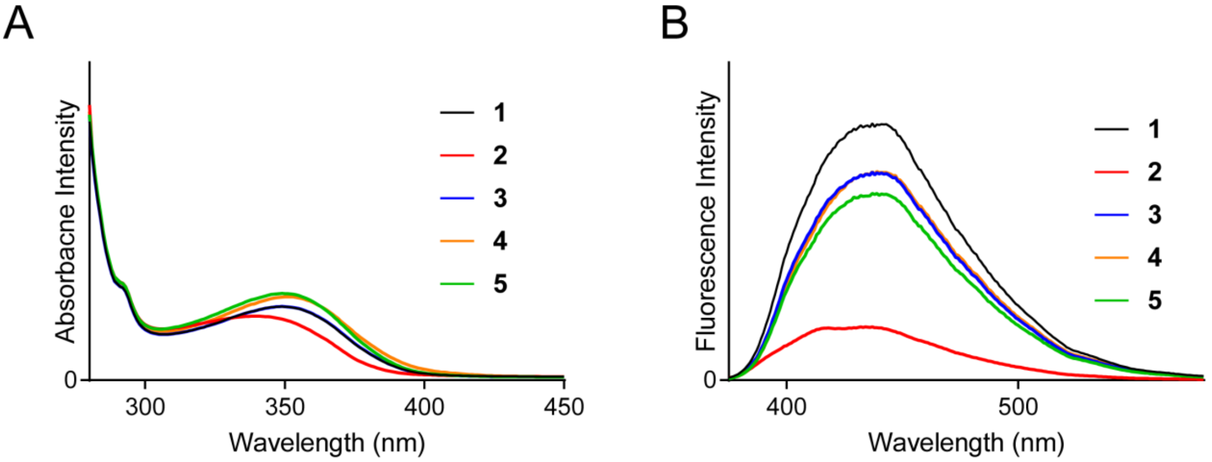

Figure S2. (A) Absorbance spectra for pyrazinones **1-5**. (B) Fluorescence spectra for pyrazinones **1-5** on excitation at 347 nm.

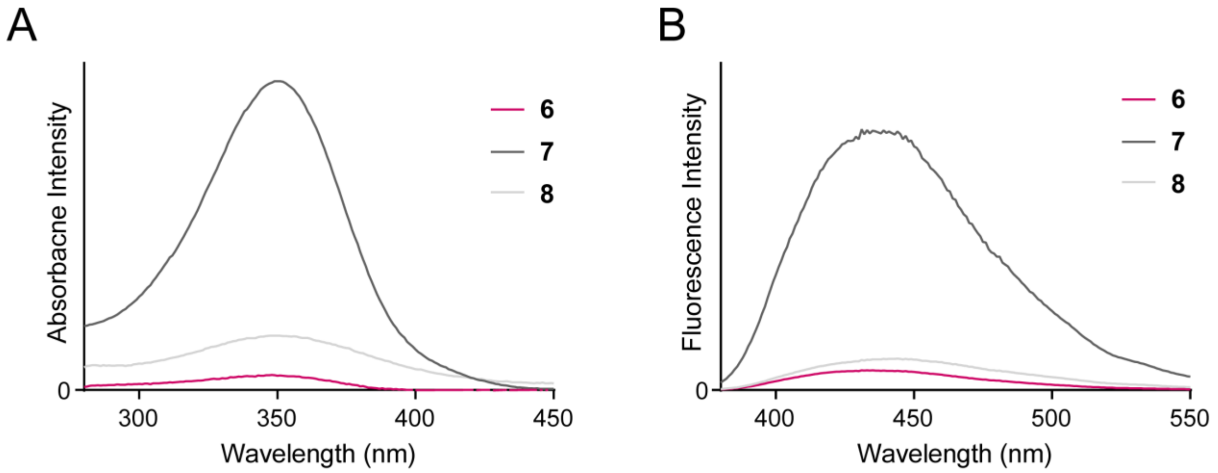

Figure S3. (A) Absorbance spectra for pyrazinones **6-8**. (B) Fluorescence spectra for pyrazinones **6**-**8** on excitation at 347 nm.
